# Blockade Antibody Responses in Human Subjects Challenged with a New Snow Mountain Virus Inoculum

**DOI:** 10.21203/rs.3.rs-3153900/v1

**Published:** 2023-09-11

**Authors:** Makoto Ibaraki, Lilin Lai, Christopher Huerta, Muktha S. Natrajan, Matthew H. Collins, Evan J. Anderson, Mark J. Mulligan, Nadine Rouphael, Christine L. Moe, Pengbo Liu

**Affiliations:** Emory University; New York University Vaccine Center, New York University; Emory University; Emory University; Emory University; Emory University; New York University Vaccine Center, New York University; Emory University; Emory University; Emory University

**Keywords:** Noroviruses, blockade antibody, Snow Mountain Virus, human challenge

## Abstract

**Background:**

Noroviruses (NoVs) are a leading cause of non-bacterial gastroenteritis in young children and adults worldwide. Snow Mountain Virus (SMV) is the prototype of NoV GII genotype 2 (GII.2) that has been developed as a viral model for human challenge models, an important tool for studying pathogenesis and immune response of NoV infections and for evaluating NoV vaccine candidates. Previous studies have identified blockade antibodies that block the binding of NoV virus-like particles (VLPs) to histo-blood group antigens (HBGAs) as a surrogate for neutralization in human Norwalk virus and GII.4 infections but little is known about SMV blockade antibodies.

**Methods:**

In this secondary data analysis study, blockade antibodies were characterized in pre-challenge and post-challenge serum samples from human subjects challenged with a new SMV inoculum. The correlation between blockade antibody geometric mean antibody titers (GMTs) and SMV-specific serum IgG/IgA GMTs were examined after stratifying the subjects by infection status. A linear mixed model was applied to test the association between HBGA blockade antibody concentrations and post-challenge days accounting for covariates and random effects.

**Results:**

Laboratory results from 33 SMV inoculated individuals were analyzed and 75.7% (25/33) participants became infected. Serum SMV-specific blockade antibodies, IgA, and IgG were all significantly different between infected and uninfected individuals beginning day 15 post-challenge. Within infected individuals, a significant correlation was observed between both IgG and IgA and blockade antibody concentration as early as day 6 post-challenge. Analysis of blockade antibody using the linear mixed model showed that infected individuals, when compared to uninfected individuals, had a statistically significant increase in blockade antibody concentrations across the post-challenge days. Among the post-challenge days, blockade antibody concentrations on days 15, 30, and 45 were significantly higher than those observed pre-challenge. The intraclass correlation coefficient (ICC) analysis indicated that the variability of blockade antibody titers is more observed between individuals rather than observations within subjects.

**Conclusions:**

These results indicate that HBGA-blockade antibody GMTs are generated after SMV challenge and the blockade antibodies were still detectable at day 45 post-challenge. These data indicate that the second generation of SMV inoculum is highly effective.

## INTRODUCTION

Human noroviruses (NoVs) are the leading cause of acute non-bacterial gastroenteritis in young children and adults globally with an estimated 70,000–200,000 deaths annually [1, 2]. NoV infection can be serious, particularly in young children, elderly, and immunocompromised people. Currently, NoVs are grouped into at least ten genogroups (GI-GX) and 49 genotypes based on the major structural protein (VP1) amino acid sequence diversity [3]. Among these genotypes, Snow Mountain virus (SMV) is the prototype of GII genogroup and genogroup II genotype 4 (GII.4) are the most prevalent strains detected in outbreaks around the world for the past two decades [4].

The human NoV genome is organized into three open-reading frames (ORF1-ORF3). ORF2 encodes the VP1 that has shell (S) and protruding (P) domains. The P domain is further divided into P1 and P2 subdomains; the P2 subdomain interacts with neutralizing/blockade antibodies and histo-blood group antigens (HBGAs) and is highly variable and evolves quickly [5, 6]. HBGAs are complex carbohydrates linked to glycoproteins or glycolipids that are present on red blood cells and mucosal epithelial cells or as free antigens in human fluids, such as saliva, intestinal contents, and human milk. NoV binds to HBGAs as receptors or co-receptors. NoV strain specific binding patterns to HBGAs have been characterized according to the ABO, secretor, and Lewis blood types of human HBGAs [7–9]. NoVs have no small animal models and it is difficult to grow human NoVs in cell lines, which challenges the study of NoV. Because of these limitations, human challenge model has been used as an important tool for studying the pathogenesis and immunology of NoV infection, and the efficacy of NoV vaccine candidates.

In previous NoVs human challenge studies, evaluation of immunity is typically limited to the use of Enzyme Immunoassay (EIA) to measure NoV-specific IgG and IgA levels in sera or saliva [10]. More recently, blockade assays are used to assess the ability of serum antibodies to block the binding of NoV virus-like particles (VLPs) to HBGAs [11–15]. These assays have been used as a surrogate for neutralization because the blockade assay is easy to perform and the neutralization antibody assay involves in complicated cell culture systems [16, 17]. While most human subjects in NoV challenge studies have pre-existing anti-NoV specific antibodies, less than 30% had pre-existing blockade antibody titers. In recent NoV challenge studies, HBGA blockade antibody titers were reported to correlate with protection against NoV-induced gastroenteritis [11, 14].

The objectives of this study were to analyze HBGA blockade antibody titers in post-challenged serum samples from in human subjects inoculated with a second generation of SMV inoculum and the magnitude and duration of SMV blockade antibodies were examined. In addition, we would like to understand what variates are associated with blockade antibodies and where the variability of blockade antibodies was derived.

## MATERIALS & METHODS

### Serum Specimens.

Serum specimens in this study were obtained from a randomized, double blind, placebo-controlled human challenge study with a new SMV inoculum that was used for studying the safety, the optimal inoculation dosage, illness, and infection of this inoculum [18]. This study was approved by the Emory Institutional Review Board and written informed consents were obtained from all subjects before enrollment. Details are available on clinicaltrials.gov (NCT 02473224) as described by Rouphael et al [18].

### Laboratory Assays.

All the assays including detection of anti-SMV IgG and IgA in serum, and detection and quantification of SMV RNA in stool, and detection of SMV blockade antibody were previously described [18]. SMV carbohydrate-binding blockade assay was developed based on previously reported Norwalk virus and Norovirus GII.4 blockade assays [19, 14]. Briefly, SMV VLP, expressed in a baculovirus expression system, were incubated with an equal volume of two-fold serially-diluted serum from the starting dilution. Simultaneously, a neutravidin-coated microplate (Piece Thermo Fisher Scientific, Rockford, IL) was coated with 2.5 μg/mL of blood type B-PAA-biotin (GlycoTech, Gaithersburg, MD) and incubated for at room temperature. After the plates were washed, the sera-VLP mixture was added to the blood type B coated plate and incubated at 4°C for 2 hours. Plates were washed again and incubated for 1 hour after the addition of SMV-specific polyclonal antibody. Horseradish peroxidase-conjugated goat anti-rabbit IgG (Sigma-Aldrich, ST. Louis, MO) was then added and the plates were incubated following by color development. OD (optical density) was measured at 450 nm wavelength using a plate reader spectrophotometer. The BT_50_ (the 50% blockade titer), defined as the reciprocal of the last dilution with OD readings less than or equal to 50% of OD of the VLP only wells, was determined for each sample. Samples with BT_50_ less than 25 were assigned a value of 12.5.

## Statistical methods

This study is a secondary data analysis. SAS version 9.4 (SAS Institute, Cary, NC, USA) was utilized to analyze the data. The geometric mean antibody titers (GMT) and geometric mean fold rise (GMFR) were calculated for both the pre-challenge and post-challenge samples. To determine the change in HBGA-blockade antibody response in post-challenge samples while accounting for the random effects by subject, a linear mixed model was applied. The model was utilized to test the association between the natural log transformed HBGA-blockade antibody concentration and day post-challenge, accounting for covariates such as age, race, and serum IgA. Unstructured correlation between different time points were obtained for each study participant and the fixed effects of inoculum dose, age, race, serum IgA and IgG, and infection status were taken into consideration when developing a model. Since the study collected samples on several post-challenge days, the days in the model were considered as an ordinal variable. The model determined whether there were any significant increases in HBGA-blockade antibody titer between pre-challenge and the four post-challenge days of the study.

## RESULTS

### Serum antibody responses in subjects challenged with SMV inoculum

#### a. Participant’s characteristics

This study only includes 33 participants from the groups 2–5 that Rouphael described [18]. Of the 33 participants, 25 were infected, determined by RT-qPCR with SMV after challenge. First, we analyzed the age, gender, race, acute gastroenteritis symptoms, and secretor status of 33 subjects. The distribution of these demographics among infected and uninfected individuals was similar. Secretor status did not statistically differ between the infected and uninfected groups. While acute gastroenteritis symptoms were frequently observed among the infected subjects, one uninfected individual also exhibited symptoms (e.g., vomiting) which was described in the previous SMV human challenge studies (Table 1) [18].

#### b. Temporal blockade antibody responses in infected and uninfected participants

**Figure 1:**
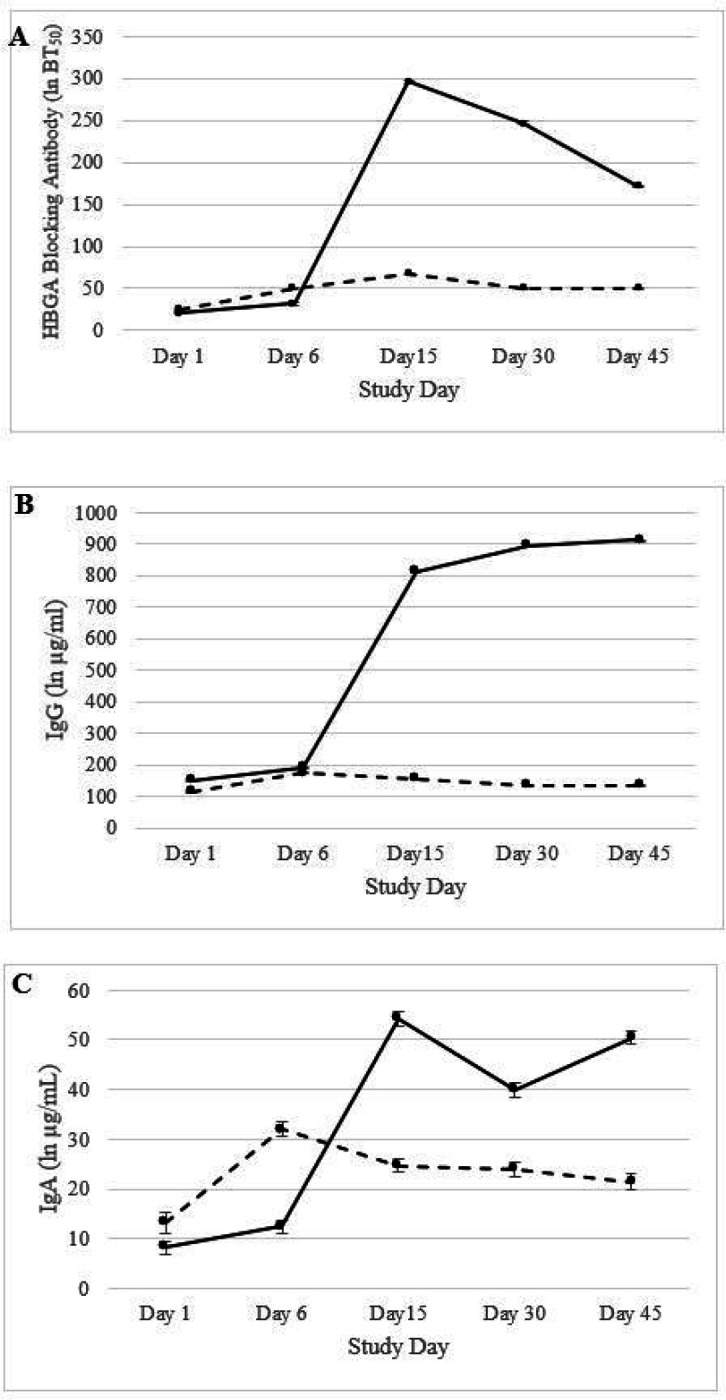
Geometric mean titer levels of pre- and post-challenge SMV specific (A) HBGA blockage antibodies, (B) serum IgG antibodies, and (C) serum IgA antibodies. Black solid lines are infected individuals while the dashed line represents uninfected individuals. The error bars indicate 95% confidence interval.

To examine whether the blockade antibody response after SMV challenge differed between infected and uninfected subjects, the GMT and GMFR of blockade antibody were compared. At days 1 and 6, both GMT and GMFR were not significantly different between infected and uninfected (P > 0.05). However, both the GMT and GMFR of blockade antibodies were significantly different between infected and uninfected individuals on days 15, 30, and 45 (Table 2). At days 15 and 30 post-challenge, 23 infected individuals had ≥ 4-fold rise in blockade antibody. At day 15, the HBGA-blockade GMTs in infected subjects reached 295.9, and then gradually declined to 247.0 and 171.0 at days 30 and 45, respectively. At day 45, 22 of the 25 infected subjects still had detectable blockade antibody titers (Table 2 and Fig. 1A).

#### c. Temporal IgG/IgA responses in infected and uninfected participants

A similar trend was observed for both serum IgG and IgA (Tables 3 and 4). For both days 1 and 6, serum IgG and IgA GMTs were relatively lower in uninfected and infected individuals. However, IgG and IgA GMTs in infected individuals sharply increased after day 6 and both IgG and IgA remained stable for days 15, 30, and 45 post-challenge (Fig. 1B and 1C).

### Correlation between serum IgG/IgA and blockade antibody titers

The correlation between blockade antibody and SMV serum IgG and IgA GMTs were examined after stratifying the subjects by infection status. No significant correlations were observed between blockade antibody and IgG/IgA among the uninfected individuals post challenge. However, IgG/IgA GMTs and blockade antibody GMTs correlated as early as day 6 for subjects who were infected and remained through day 45. Starting from day 15 post-challenge, the correlation coefficients between blockade antibody and IgG/IgA GMTs were significantly stronger and the trend slightly declined afterwards (Table 5).

### Association between blockade antibody and other covariates

The blockade antibody GMTs was examined using a linear mixed model that accounted for correlation within repeated subject measurements over time. The mixed model concluded that compared to the pre-challenge day (day 1), post-challenge days 15, 30, and 45 had significantly higher HBGA-blockade antibody GMTs with P values of < 0.001, < 0.001, and 0.001, respectively. SMV-specific serum IgA was also associated with HBGA-blockade antibody GMTs (Table 6). The other variables examined in the linear mixed model, which includes inoculum dose, age, and race, did not correlate with HBGA-blockade antibody levels.

The intraclass correlation coefficient (ICC) in the linear mixed model also examined the question of how much variability of HBGA-blockade antibody titer within individuals to the variability across individuals after controlling for the baseline covariates. The ICC for this model (data not shown) was 0.45 (95% CI: 0.29, 0.58), this poor correlation indicating that the variability of blockade antibody titer is observed between individuals rather than within subjects.

## DISCUSSION

In early controlled human challenge studies and outbreak investigations, NoV immunity was assessed by serum IgG or IgM responses [20, 10, 21]. Later on, several studies successfully detected mucosal IgA in fecal or saliva samples and demonstrated a correlation between memory IgA response and protection from NoV infection [22–24]. In recent years, blockade antibody was developed and detected in serum samples as a surrogate for assessing neutralizing antibody response after NoV infections [11, 13–15]. NoV strains specific binding patterns to HBGAs have been characterized in several *in vitro* studies according to the ABO, secretor, and Lewis blood types of human HBGAs [7–9]. This association was first demonstrated in an early human challenge study with Norwalk virus, showing that only secretor-positive subjects became infected and secretor-negative subjects could not be infected with experimental NoV challenge [22]. Subsequent human challenge and laboratory studies further demonstrated the importance of secretor status on the susceptibility to NoV GII.4 strains [25] but not to SMV [26, 18]. This mechanism led to the development of a NoV blockade antibody assay that uses serum from infected human subjects to block the binding of NoV VLPs to HBGAs, which serves as a surrogate for neutralizing activity that is not easy to determine [11, 15]. Atmar et al.,[17] reported that HBGA-blockade antibody titers highly correlated with the neutralizing antibody titers among 24 healthy participants who received a bivalent GI.1 and GII.4 NoV vaccine, particularly for the homologous variant that blockade antibody titers to the GI.1 highly correlated with the neutralizing antibody response measured using GI.1 and same results were for GII.4. This result suggests that HBGA-blockade antibody levels are a surrogate for neutralization antibodies. Previous studies have investigated the roles of serum blockade antibodies in vaccinated subjects [27, 14], experimentally challenged individuals [11], naturally infected travelers [21], and young children [28]. Atmar et al.,[14] demonstrated that pre-challenge HBGA-blockade antibody levels were associated with a lower risk of norovirus infection and illness in subjects administered two doses of bivalent GI.1/GII.4 vaccine and subsequently challenged with a NoV GII.4 strain. This association was confirmed in a human challenge study [11] and naturally NoV infected children in strain-specific manner [28] but other studies have not identified this association [29, 13].

It is important to understand the breadth of seroconversion of blockade antibodies and how long the HBGA-blockade antibody lasts after infection. Frenck et al., indicated that 23 secretor positive healthy adults challenged with 5 × 10^4^ reverse-transcription polymerase chain reaction (RT-PCR) units of GII.4 NoV, 21 (91.3%) developed > 50 BT_50_ HBGA-blockade antibody levels on day 30 after challenge [30]. In human volunteers immunized with a multivalent NoV VLPs (genotypes GI.1/GII.4), HBGA-blockade antibody titers against both vaccine strains were elicited in 80% (8/10), 100% (10/10), and 55.6% (5/9) of subjects for GI.1 genotype and 60% (6/10), 70% (7/10), and 11.1% (1/9) for GII.4 strain at days 7, 35, and 180 [15]. In our SMV human challenge study, 23 individuals infected with SMV still had ≥ 4-fold rise in blockade antibody and the GMTs peaked at day 15. At day 45, 22 of 25 infected subjects still had detectable blockade antibody titers. The blockade antibody positivity and duration from our study are generally consistent with reports from other studies. However, from all of these studies, it is unclear how long the HBGA-blockade antibody lasts since the longest follow-up time was only up to day 180 after virus challenge. To answer this question, Simmons et al., predicated using a mathematical modeling that immunity to after NoV infection lasted approximately between 4.1 and 8.7 years [31]. If correct, this duration of protection is longer than previously estimated, and could be crucial for NoV vaccine development and clinical trials for evaluation of vaccine efficacy.

The primary limitation of this study is that we were unable to understand the association between pre-challenge blockade antibody and post-challenge protection from infection and illness since only few subjects had measurable level of blockade antibody before challenge. In addition, from data analysis standpoint, sample size in this study is relatively small. Of the 44 enrolled individuals, only 33 subjects were included in this study and had completed SMV blockade antibody results for analysis. Because of the small sample size, the statistical results obtained from this study may be biased.

Challenge of the second generation of SMV inoculum in human subjects in this study induced HBGA antibodies that remained elevated through day 45 post-challenge. These data indicate that the second generation of SMV inoculum is highly effective and can be used in the evaluation of NoV vaccine candidates as well as study NoV pathogenesis and immunity in humans.

## Figures and Tables

**Table 1.** Characteristics of SMV challenged subjects stratified by infection status

Characteristic	Infected* (n=25)	Uninfected (n=8)	*P* value
Age (year) (SD)	33.0 (9.4)	33.8 (10.2)	0.908^a^
Female	11 (44.0%)	2 (25.0%)	0.403^b^
Race			
White	8 (32.0%)	1 (12.5%)	0.442^c^
Black	15 (60.0%)	7 (77.5%)	
Multiple	2 (8.0%)	0	
AGE symptoms			
Vomit	11 (44.0%)	1 (11.1%)	0.114^b^
Diarrhea	8 (32.0%)	0 (0%)	0.077^b^
Secretor Status			
Positive	18 (72.0%)	7 (87.5%)	0.605^b^
Negative	7 (28.0%)	1 (12.5%)	

AGE: acute gastroenteritis

SD: standard deviation

* Infected was defined as SMV RNA positive in any post-challenge stool sample detected by RT-qPCR and/or serum IgG >4-fold rise between post- vs. pre-sample.

a Two-sample *t* test P value

b Fisher’s exact P value

c Pearson ^2^ P value

**Table 2:** Anti-SMV HBGA blockade antibody response to SMV challenge in pre- and post- samples

	Infection Status^a^		
	Infected (n = 25)	Uninfected (n = 8)	P value^c^
Day 1^b^			
N	25	8	
GMT (95% CI)	20.6 (16.0, 26.6)	25.0 (17.2, 36.4)	0.257
GMFR (95% CI)	0	0	
Day 6			
N	23	6	
GMT (95% CI)	31.8 (21.1, 48.0)	50.0 (21.9, 114.4)	0.201
GMFR (95% CI)	1.6 (1.2, 2.0)	1.6 (0.9, 3.0)	0.935
Day 15			
N	23	6	
GMT (95% CI)	295.9 (141.4, 619.5)	67.3 (22.4, 202.5)	0.031
GMFR (95% CI)	14.6 (7.0, 30.7)	2.21 (0.9, 5.6)	0.018
Day 30			
N	23	5	
GMT (95% CI)	247.0 (121.6, 501.5)	50.0 (13.6, 183.7)	0.034
GMFR (95% CI)	12.2 (6.0, 25.0)	1.6 (0.5, 5.2)	0.011
Day 45			
N	22	5	
GMT (95% CI)	171.0 (93.8, 311.1)	50.0 (17.9, 139.9)	0.037
GMFR (95% CI)	8.5 (4.7, 15.6)	1.59 (0.7, 3.8)	0.010

a Infection was defined as SMV RNA positive in any post-challenge stool sample detected by RT-qPCR.

b Day 1 was pre-challenge

c Kruskal-Wallis P value indicating probability of statistically significant difference in GMT and GMFR between infected and uninfected subjects post challenge

Abbreviation: CI - confidence interval, GMT - geometric mean antibody titers, GMFR - geometric mean fold rise

**Table 3:** SMV-specific serum IgG response to SMV challenge in pre- and post- samples

	Infection Status^a^		
	Infected (n = 25)	Uninfected (n = 8)	P value^b^
Day 1^c^			
N	25	8	
GMT (95% CI)	149.7 (105.1, 213.3)	115.0 (50.9, 259.8)	0.301
GMFR (95% CI)	0	0	
Day 6			
N	23	6	
GMT (95% CI)	190.2 (129.3, 279.7)	175.0 (77.5, 395.4)	0.364
GMFR (95% CI)	1.3 (1.1, 1.5)	1.2 (0.8, 1.6)	0.333
Day 15			
N	23	6	
GMT (95% CI)	812.3 (454.6, 1451.4)	154.4 (69.5, 343.0)	0.001
GMFR (95% CI)	5.5 (3.0, 9.9)	1.0 (0.8, 1.3)	0.008
Day 30			
N	23	5	
GMT (95% CI)	895.5 (537.7, 1491.4)	135.2 (56.6, 323.0)	0.001
GMFR (95% CI)	6.0 (3.4, 10.8)	1.0 (0.8, 1.1)	0.006
Day 45			
N	22	5	
GMT (95% CI)	912.3 (554.3, 1501.4)	133.7 (62.2, 287.3)	0.001
GMFR (95% CI)	6.1 (3.5, 10.7)	0.9 (0.6, 1.4)	0.004

a Infection defined as SMV excretion in stool detected through RT-qPCR at any time after challenge through Day 45

b P value obtained from Kruskal-Wallis test

c Day 1 denotes pre-challenge

Abbreviation: CI - confidence interval, GMT - geometric mean antibody titers, GMFR - geometric mean fold rise

**Table 4:** SMV-specific serum IgA response to SMV challenge in pre- and post- samples

	Infection Status^a^		
	Infected (n = 25)	Uninfected (n = 8)	P value^b^
Day 1^c^			
N	25	8	
GMT (95% CI)	8.1 (4.9, 13.4)	13.8 (2.6, 70.5)	0.223
GMFR (95% CI)	0	0	
Day 6			
N	25	6	
GMT (95% CI)	12.4 (6.1, 25.2)	34.1 (12.8, 76.5)	0.165
GMFR (95% CI)	1.5 (0.8, 2.9)	1.3 (0.7, 1.6)	0.116
Day 15			
N	25	6	
GMT (95% CI)	54.2 (25.2, 116.7)	26.8 (10.1, 59.9)	0.062
GMFR (95% CI)	6.7 (2.7, 16.5)	1.9 (0.4, 1.4)	0.025
Day 30			
N	25	5	
GMT (95% CI)	39.9 (18.4, 86.3)	25.5 (8.9, 69.9)	0.231
GMFR (95% CI)	4.9 (2.0, 11.9)	1.0 (0.7, 1.3)	0.012
Day 45			
N	22	5	
GMT (95% CI)	50.4 (34.4, 73.8)	23.4 (7.9, 68.5)	0.043
GMFR (95% CI)	5.5 (3.3, 9.2)	0.9 (0.5, 1.4)	0.012

a Infection defined as SMV excretion in stool detected through RT-qPCR at any time after challenge through Day 45

b P value obtained from Kruskal-Wallis test

c Day 1 denotes pre-challenge

Abbreviation: CI – confidence interval, GMT – geometric mean antibody titers, GMFR – geometric mean fold rise

**Table 5.** Correlation between log-transformed IgG and IgA titers and log-transformed HBGA blockade antibody titers

	SMV serum IgG		SMV serum IgA	
	Infected (n = 25)	uninfected (n = 8)	Infected (n = 25)	Uninfected (n = 8)
Day 1				
N	25	8	25	8
R^2^	0.19	0.39	0.30	0.24
P value	0.028	0.09	0.005	0.18
Day 6				
N	23	6	23	7
R^2^	0.48	0.02	0.40	0.17
P value	0.0002	0.81	0.001	0.36
Day 15				
N	23	6	23	23
R^2^	0.75	0.01	0.77	0.03
P value	< 0.0001	0.87	< 0.0001	0.72
Day 30				
N	23	5	23	6
R^2^	0.71	0.10	0.68	0.01
P value	< 0.0001	0.61	< 0.0001	0.89
Day 45				
N	22	5	22	6
R^2^	0.57	0.04	0.61	0.02
P value	< 0.0001	0.75	< 0.0001	0.77

R^2^: R-squared (0–1) that measures the linear association between IgG/IgA and blockade antibodies

**Table 6:** Association between covariates and HBGA-blockade antibody titer accounting for time-points among study subjects

Variables	β estimates^a^	95% CI^b^	P Value
Visit Days (vs. Day 1)			
Day 6	−0.004	−0.49, 0.49	0.99
Day 15	1.49	0.79, 2.11	< 0.001
Day 30	1.54	0.79, 1.98	< 0.001
Day 45	1.15	0.49, 1.65	0.001
Inoculum dose	0.002	−0.0001, 0.001	0.113
Age	0.008	−0.03, 0.04	0.673
Race (vs. White)			
Black	−0.88	−1.47, −0.29	0.006
Multiple	−0.35	−1.57, 0.88	0.565
In transformed IgA	0.6	0.41, 0.79	< 0.001

a β denotes coefficient

b CI is abbreviation for confidence interval
